# Low Serum Complement C3 Levels at Diagnosis of Renal ANCA-Associated Vasculitis Is Associated with Poor Prognosis

**DOI:** 10.1371/journal.pone.0158871

**Published:** 2016-07-08

**Authors:** Jean-François Augusto, Virginie Langs, Julien Demiselle, Christian Lavigne, Benoit Brilland, Agnès Duveau, Caroline Poli, Alain Chevailler, Anne Croue, Frederic Tollis, Johnny Sayegh, Jean-François Subra

**Affiliations:** 1 LUNAM Université, Angers, France; 2 Université Angers, CHU Angers, Service de Néphrologie-Dialyse-Transplantation, Angers, France; 3 Université Angers, CHU Angers, Service de Médecine Interne, Angers, France; 4 Université d’Angers, CHU Angers, Laboratoire d’Immunologie, Angers, France; 5 Université Angers, CHU Angers, Département de Pathologie Cellulaire et Tissulaire, Angers, France; 6 Diaverum Dialysis Center, Angers, France; Nippon Medical School Graduate School of Medicine, JAPAN

## Abstract

**Background:**

Recent studies have demonstrated the key role of the complement alternative pathway (cAP) in the pathophysiology of experimental ANCA-associated vasculitis (AAV). However, in human AAV the role of cAP has not been extensively explored. In the present work, we analysed circulating serum C3 levels measured at AAV onset and their relation to outcomes.

**Methods:**

We conducted a retrospective observational cohort study including 45 consecutive patients with AAV diagnosed between 2000 and 2014 with serum C3 measurement at diagnosis, before immunosuppressive treatment initiation. Two groups were defined according to the median serum C3 level value: the low C3 group (C3<120 mg/dL) and the high C3 level group (C3≥120 mg/dL). Patient and renal survivals, association between C3 level and renal pathology were analysed.

**Results:**

Serum complement C3 concentration remained in the normal range [78–184 mg/dL]. Compared with the high C3 level, the patients in the low C3 level group had lower complement C4 concentrations (P = 0.008) and lower eGFR (P = 0.002) at diagnosis. The low C3 level group had poorer patient and death-censored renal survivals, compared with the high C3 level group (P = 0.047 and P = 0.001, respectively). We observed a significant negative correlation between C3 levels and the percentage of glomeruli affected by cellular crescent (P = 0.017, r = -0.407). According to the Berden et al renal histologic classification, patients in the crescentic/mixed category had low C3 levels more frequently (P<0.01). Interestingly, we observed that when patients with the crescentic/mixed histologic form were analysed according to C3 level, long term renal survival was significantly greater in the high C3 level group than in the low C3 level group (100% vs 40.7% at 6 years, p = 0.046). No relationship between serum C4 and renal outcome was observed.

**Conclusion:**

A Low C3 serum level in AAV patients at diagnosis is associated with worse long-term patient and renal survival.

## Introduction

Anti-neutrophil cytoplasmic antibody (ANCA)-associated vasculitides are life threatening autoimmune diseases characterised by necrotising inflammation of small to medium-sized vessels [1–3] and the detection of ANCAs in serum. Three entities are differentiated based on clinical and pathological criteria, with overlapping clinical spectra: microscopic polyangiitis (MPA), granulomatosis with polyangiitis (GPA), and eosinophilic granulomatosis with polyangiitis (EGPA) [4]. AAV is frequently a multi-system disease with a predilection for affecting the respiratory tract and kidney parenchyma [2]. When the kidneys are involved, AAV typically gives rise to rapidly progressive renal failure and necrotising crescentic glomerulonephritis (NCGN) [5]. In contrast to immune-complex associated diseases (i.e., systemic lupus erythematosus or IgA vasculitis), the hallmark of AAV is the lack or paucity of immunoglobulin and complement deposits [4, 6], suggesting that the complement system may not play an important role in the pathophysiology of human AAV.

The understanding of human AAV pathophysiology has greatly benefited from discoveries made in murine MPO-ANCA vasculitis models [7, 8]. In this model, MPO-ANCAs are induced in MPO-deficient mice and transferred into wild-type mice, resulting in the development of NCGN. Using this model, Xiao et al were the first to demonstrate that the complement alternative pathway (cAP) plays a crucial role in experimental vasculitis [9]. Indeed, they applied the MPO-ANCA vasculitis model to mice decomplemented with cobra venom factor, and to C5-, C4- and Factor B deficient mice. In mice with impaired cAP (C5 or Factor B deficient mice) the development of NCGN was completely abrogated, in contrast to C4-deficent mice with defective classical complement pathway. The pathophysiologic importance of the cAP was further evidenced by demonstrating the pivotal role of the anaphylatoxin C5a and its receptor (C5aR/CD88) for the mediation of NCGN [10–12].

In humans, AAV-NCGM is classically considered as a pauci-immune entity. However, Haas et al in 2004, were the first to show a high frequency of, usually mild deposits of immunoglobulins or complement components in renal biopsies of AAV patients by using direct immunofluorescence and electron microscopy [13]. Further data came from the study performed by Chen et al showing C3c deposition in one third of renal biopsies from 112 AAV patients [14]. The first evidence of cAP activation in human AAV came from Gou et al, who showed elevated serum factor Bb, a specific compound of the cAP, in active AAV patients, at higher levels than in remission patients and SLE active patients [15]. Most recently, factor Bb, C3d and properdine deposits in AAV kidney biopsies have been reported, thus supporting cAP implication in renal injury [16–18]. Also supporting an important role of complement system in human AAV, CCX168, the anti-C5a receptor inhibitor, showed promising results on renal disease parameters in an ongoing phase II trial (ClinicalTrials.gov, identifier: NCT02222155). Altogether, these data not only support the fact that the complement system is involved in human AAV pathophysiology, but also that activation might be mediated by cAP activation.

In the present study, we analysed serum complement C3 levels in 45 newly diagnosed AAV patients and studied the relationship between C3 levels, renal histologic findings, and patient prognosis.

## Patients & Methods

### Study population

All patients, positive for either PR3- or MPO-ANCAs, admitted to the Nephrology department of the University Hospital of Angers between January 2000 and December 2014 were considered for inclusion in the study. During the study period, 55 consecutive AAV patients, positive for PR3- or MPO-ANCAs, were admitted with new onset AAV. Patients with ANCA-negative AAV were excluded from the present study. Of the 55 consecutive patients, 45 patients had serum complement C3 and C4 determination before treatment initiation and entered the study. The patients were followed until death or end of follow-up. The study protocol was specifically approved by the Ethics Committee of Angers University Hospital (CE 2011/06, S1 File). The Ethics Committee approved the analysis of files from ANCA associated vasculitis patients of the Nephrology department of Angers University Hospital. Given that data were extracted from patients files, a written informed consent was not required. Patients records and related informations were anonymized in databases before author's access

### Data collection

All the data were collected retrospectively following individual screening of the patients' medical records. The following data were retrieved: age, gender, weight, and significant past medical history. The nature and type of injuries to the affected organs at presentation were listed. The activity of ANCA-associated vasculitis was determined using the Birmingham Vasculitis Activity Score (BVAS) 2003 [19]. The C3 and C4 levels were assessed by nephelometry (BNP Prospec, Siemens Behring) with reference ranges supplied by the vendor (normal range 75–175 mg/dL and 14–40 mg/dL, respectively). After venipuncture, clot was allowed to occur in four hours at room temperature and removed by centrifugation at 3000 g. Sera were stored no more than two days at +4°C before analysis. The other following biological data were retrieved: serum albumin, haemoglobin, and C-reactive protein level. Glomerular filtration rate on admission was calculated using the 4-variable Modification of Diet in Renal Disease (MDRD) study equation [20].

### Definitions

Patient folders were analysed and AAV subtype (GPA, MPA and EGPA) was determined according to the European Medicines Agency (EMEA) vasculitis classification algorithm [21]. Renal disease was based on clinical data (active urinary sediment, proteinuria and impaired renal function) and/or renal biopsy. Renal death was defined as the need for renal replacement therapy (RRT) for more than 3 months. The need for RRT at diagnosis was defined as the need for RRT during the first hospital stay.

### Histologic studies

A renal biopsy was available for 34 of the 45 patients. Renal biopsy specimens were routinely assessed by light microscopy and direct immunofluorescence. For light microscopy, formalin-fixed and paraffin-embedded sections were stained with periodic acid-Schiff, haematoxylin and eosin and Masson trichrome. For the direct immunofluorescence study, IgG, IgM, IgA, C3 and C1q antibodies were used to detect respective deposits. All renal biopsy slides were reviewed by an expert pathologist who was blinded to the patients’ medical records. Light microscopy slides were classified into four categories according to the Berden et al classification (focal, crescentic, mixed and sclerotic) [22]. For the direct immunofluorescence study, staining were done on fresh frozen renal tissue immediately after renal biopsy using the following FITC conjugated antibodies: polyclonal rabbit anti-human IgA, IgG, IgM, C3 and C1q (Dako, Denmark A/S). Slides were analysed using DMRB Leica IF microscope. The intensity of fluorescence was graded as follows: “0” (negative), “+” (slightly positive), “++” (moderately positive), “+++” (intensely positive).

### Statistical analysis

Two groups were determined and compared according to the median level of serum complement C3 observed in the whole cohort. Hence, patients with serum C3 level < 120 mg/dL were classified in the low C3 group and patients with C3 level ≥ 120 mg/dL in the high C3 group.

Continuous variables are presented as means ± SD and minimum/maximum value. Categorical variables are presented as the absolute value and percentage. Differences between groups were analysed using the χ^2^ test (or Fisher exact test) for categorical variables and the Mann-Whitney U test for continuous variables. The Kaplan-Meyer method was used to analyse group survival. A log-rank test was used to compare the survival curves. All the statistical tests were performed to the two-sided 0.05 level of significance. Statistical analysis was performed using SPSS software® 23.0 for Mackintosh and Graphpad Prism^®^.

## Results

### Clinical and biological characteristics of the population at AAV presentation

The study included 45 consecutive patients for whom serum complement C3 was measured at AAV diagnosis, before initiation of immunosuppressive treatment. Mean follow-up of the patients was 54.6±49.1 [0.3–171] months. The patient characteristics at AAV diagnosis are summarised in Table 1. The patients were predominantly male with a mean age of 64.1 years. GPA was diagnosed according to the EMEA algorithm in 60.0% (27/45) of the patients and MPA in 40% (18/45) of the patients (7 with renal-limited vasculitis). ANCAs were p-ANCAs in 64.5% of patients and MPO specific in 62.3% of cases. Mean BVAS was 16.2±4.4. Ear-nose-throat, lung and renal involvements were the most common organ involvements at presentation. All patient had renal disease and 31.1% of patients required renal replacement therapy at diagnosis. Mean serum creatinine and eGFR were 385.9±401.2 μmol/L and 34.7±32.7 mL/min/1.73 m^2^ at presentation, respectively. Mean serum C3 and C4 levels were 125.4 and 26.2 mg/dL respectively.

**Table 1:** Clinical and biological presentation at AAV diagnosis.

	n = 45
**Baseline characteristics**	
Sex (M/F)	26/19
Age (years)	64.1 ± 14.3 [18–86]
Weight (kg)	72.5 ± 14.9 [50–107]
Hypertension, n (%)	24 (53.3)
Diabetes mellitus, n (%)	6 (13.3)
**ANCA-associated vasculitis characteristics**	
**Diagnosis**, n (%)	
GPA/MPA, n (%)	27 (60.0) / 18 (40.0)
**ANCA type**	
c-ANCA/p-ANCA, n (%)	16 (35.5) / 29 (64.5)
PR3-ANCA/MPO-ANCA, n (%)	17 (37.7) / 28 (62.3)
**Complement component level**, mg/dL	
C3 level	125.4 ± 25.2 [78–184]
C4 level	29.1 ± 9.6 [10–58]
**BVAS**	16.2 ± 4.4 [12–32]
**Organ involvement**, n (%)	
Cutaneous signs	8 (17.7)
Ear, nose, throat	19 (42.2)
Heart	2 (4.4)
Digestive	3 (6.7)
Lung	17 (37.8)
Alveolar hemorrhage	3 (6.7)
Renal	45 (100)
Serum creatinine	385.9 ± 401.2 [49–1906]
eGFR	34.7 ± 32.7 [2.2–115.4]
Renal replacement therapy at diagnosis	14 (31.1)
Neurological	4 (8.9)
**Immunosuppressive treatment**	
Steroids—Cyc	34 (75.5)
Steroids—PE—Cyc	11 (24.5)

### Presentation according to serum C3 complement levels at AAV diagnosis

We first analysed the association between serum complement C3 level and features of AAV patients at presentation. For this purpose, we determined two groups of AAV patients according to the median C3 concentration. Hence the low C3 group included patients with C3<120 mg/dL and the high C3 group patients with C3 ≥120 mg/dL. Of note, complement C3 level remained in the normal range for all patients.

Compared with the high C3 level group, patients in the low C3 group tended to be older (p = 0.150). There was no difference between groups according to ANCA type at IIF and ANCA specificity (p = 0.256 and p = 0.420, respectively). Serum complement C4 level was significantly lower in the low C3 level group compared with the high C3 level group (p = 0.008). No difference was observed in the frequency of anti-nuclear antibodies, serum albumin and haemoglobin levels.

Disease activity assessed by BVAS was comparable in both groups. Serum creatinine was higher and eGFR was lower in the low C3 group compared with the high C3 group (p = 0.008 and p = 0.002, respectively). No significant differences between groups were observed according to other organ involvement at AAV diagnosis. These results are reported in Table 2.

**Table 2:** Comparison of clinical and biological features according to serum C3 levels.

	Low C3 group	High C3 group	*P*
	n = 22	n = 23	
**Baseline characteristics**			
Sex (M/F), n (%)	11/11	15/8	0.302
Age (years)	67.2 ± 13.4 [28–83]	61.0 ± 14.8 [18–77]	0.150
Weight (kg)	70.9 ± 15.1 [51–102]	73.9 ± 14.9 [50–107]	0.526
Hypertension, n (%)	15 (68.2)	9 (39.1)	0.051
Diabetes mellitus, n (%)	3 (13.6)	3 (13.0)	1.000
**Biological characteristics**			
Complement component level, mg/dL			
C3 level	105.4 ± 11.1 [78–120]	144.5 ± 19.2 [121–184]	**<0.0001**
C4 level	25.3 ± 7.3 [10–39]	32.7 ± 10.2 [14–58]	**0.008**
Antinuclear antibodies (>1/200)	7 (31.8)	3 (13.0)	0.129
Serum albumin, g/L	30.2 ± 7.0 [22–42]	29.8 ± 6.3 [17–40]	0.873
C-reactive protein, mg/dL	75.1 ± 55.6 [8–167]	123.2 ± 96.0 [10–297]	0.057
Hemoglobin, g/dL	9.8 ± 1.7 [7–12.2]	9.2 ± 1.9 [5.7–11.1]	0.393
Lactate deshydrogenase (UI/L)	384.0 ± 141 [151–693]	269.5 ± 81 [143–459]	**0.013**
**ANCA-associated vasculitis characteristics**			
**Diagnosis**, n (%)			
GPA/MPA	12 (54.5) / 10 (45.5)	15 (65.2) / 8 (34.8)	0.465
c-ANCA / p-ANCA	6 (27.2) / 16 (72.8)	10 (43.5) / 13 (56.5)	0.256
PR3/MPO-ANCA	7 (31.8) / 15 (68.2)	10 (43.5) / 13 (56.5)	0.420
**BVAS**	16.0 ± 4.7 [12–32]	16.5 ± 4.1 [12–27]	0.719
**Organ involvement**, n (%)			
Cutaneous signs	4 (18.2)	4 (17.4)	1.000
Ear, nose, throat	7 (31.8)	12 (52.2)	0.167
Heart	1 (4.6)	1 (4.3)	1.000
Digestive	1 (4.5)	2 (8.7)	1.000
Lung	8 (36.4)	9 (39.1)	0.848
Alveolar hemorrhage	1 (4.5)	2 (8.7)	1.000
Renal	22 (100)	22 (95.7)	1.000
Serum creatinine, μmol/L	543.6 ± 491.5 [67–1906]	235.0 ± 206.7 [49–700]	**0.008**
eGFR, mL/min/1.73 m^2^	20.1 ± 22.0 [2.2–96.6]	48.6 ± 35.5 [5.4–115.4]	**0.002**
Proteinuria/creatininuria (g/g)	4.3 ± 3.3 [1.2–16.0]	2.5 ± 2.4 [0.2–9.7]	0.059
Renal replacement therapy at diagnosis, n (%)	9 (40.9)	5 (21.7)	0.164
Neurological	1 (4.5)	3 (13.0)	1.000
Immunosuppressive treatment			
Steroids—Cyc	15 (68.2)	19 (82.6)	0.314
Steroids—PE—Cyc	7 (31.8)	4 (17.4)	0.314
Patient’s follow-up* (months)	45.4 ± 47 [0.1–173]	63.4 ± 50 [7.1–163]	0.156

* until death or last visit.

### Association between serum complement C3 and AAV prognosis

**Fig 1:**
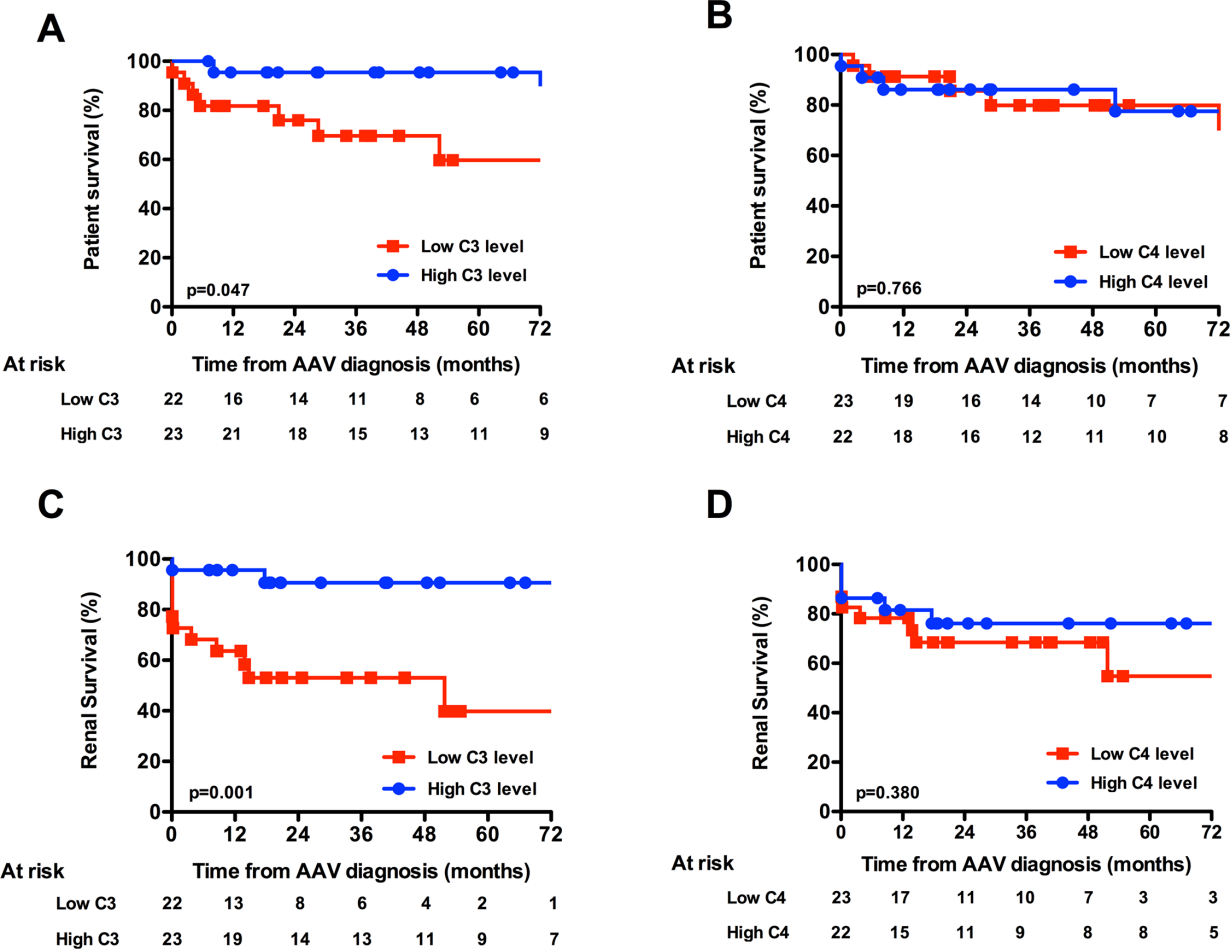
Patient and death-censored renal survivals according to C3 and C4 levels. (A) Patient survival according to C3 level and (B) C4 level. (C) Death-censored renal survival according to C3 lavel and (D) C4 level.

We next analysed the patient and death-censored renal survivals according to C3 and C4 level (Fig 1A–1D). We observed that patients in the low C3 group had significantly poorer long-term survival compared with the high C3 level group (p = 0.047). In a similar manner, renal survival was significantly poorer in the low C3 group (p = 0.001). When patients were classified according to ANCA specificity, PR3-ANCA patients with low C3 serum levels had significantly poorer renal survival than those with high C3 levels (p = 0.003) (S1A Fig). Classification according to MPO-ANCA showed similar tendency (p = 0.095) (S1B Fig). When analysed in a logistic regression model, low C3 level appeared as a significant risk factor of renal death (OR = 7.1, [1.2–41.6], p = 0.03), after adjustment on renal function at diagnosis (creatinine >150 μmol/L, OR = 3.5, [0.22–36.2], p = 0.298).

We did not observe any difference in the rate of dialysis interruption according to median C3 levels in patients that required renal replacement therapy at diagnosis (S2A Fig), even though the C3 level of patients that remained dialysis-dependant was significantly lower than patients with dialysis interruption (S2B Fig). Interestingly, all patients that recovered renal function were classified in the high C3 level group.

In contrast, patient and renal survival was no different when patients were analysed according to serum complement C4 levels (p = 0.766 and p = 0.380, respectively). Moreover, we did not observe any association between serum complement C3 levels and the risk of AAV relapse or the occurrence of infectious events (data not shown).

### Association between serum complement C3 levels and histological kidney involvement

Given the higher severity of renal disease in the low C3 group, we next analysed the relationship between C3 level and renal histologic lesions. We performed this analysis in 34 out of 45 patients for whom a renal biopsy was performed at diagnosis. These 34 patients were equally distributed in respect to C3 level (17 patients within each C3 group).

**Fig 2:**
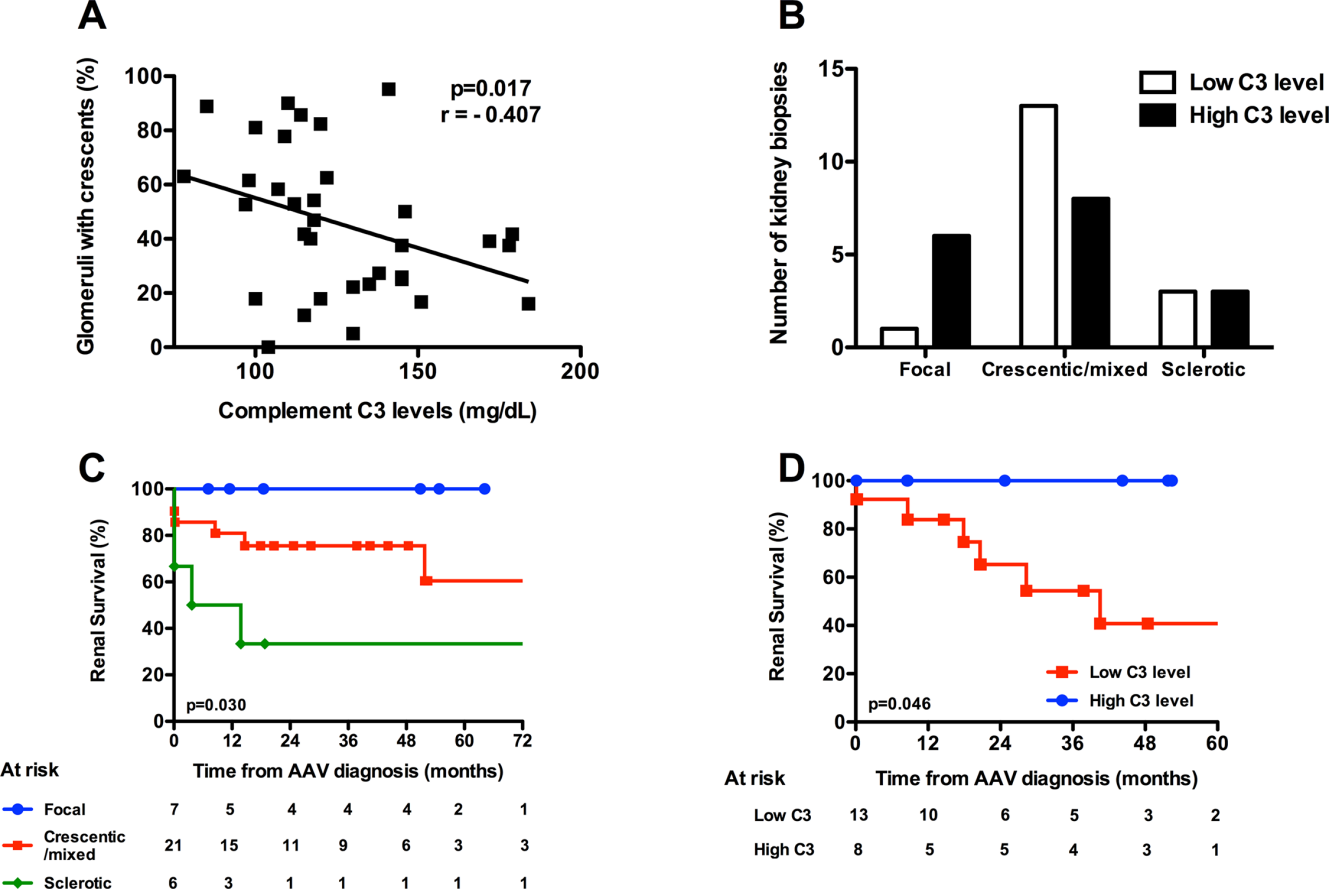
Relationship between C3 level and renal histologic involvement. (A), Correlation between C3 level and % of glomeruli with cellular crescents. Analysis was done using Spearman’s correlation test. (B), Kidney histological involvement according to Berden’s classification and C3 levels. (C), Renal survival according to histological involvement, and (D) according to C3 levels in patients with crescentic/mixed involvement.

First, we observed a significant negative correlation between serum C3 levels and the percentage of glomeruli affected by cellular crescents (Fig 2A; p = 0.017, r = -0.407). According to the Berden et al classification, 7 (20.6%), 21 (61.8%) and 6 (17.6%) biopsies were classified in the focal, crescentic/mixed and sclerotic categories respectively. When the biopsies were analysed according to histologic categories, we observed that patients in the crescentic/mixed category tended to be more frequently classified in the low C3 group (p = 0.07, Fig 2B). We also observed that overall 80% of the patients had immunoglobulin and/or complement deposits in the immunofluorescence study of the renal biopsy, although deposits were graded as low intensity (grade 1+ on a scale of 0 to 4+). We did not observe any differences in immunoglobulin or complement deposits when the biopsies were analysed according to C3 levels (S3 Fig).

According to the literature, and compared with patients in the focal and sclerotic categories, patients in the crescentic and mixed histologic categories had intermediate renal prognoses (Fig 2C) [22]. We therefore wondered if C3 level might help to differentiate renal prognosis for patients in the crescentic/mixed renal categories. We observed that patients with crescentic or mixed renal involvement and high C3 levels had excellent death-censored renal survival (Fig 2D). In opposition, patients with crescentic/mixed renal involvement and low serum C3 levels had poorer renal survival (100% vs 40.7% at 6 years, p = 0.046).

## Discussion

The present study highlights an association between serum complement C3 level measured at AAV diagnosis and the risk of end-stage renal disease and mortality. The histologic analysis showed that C3 level was associated with the intensity of glomerular extracapillary proliferation, leading to the identification of a subset of patients with worse renal prognosis. Thus, these results not only support the implication of the complement system in AAV renal involvement, but also imply that C3 level may represent a potential biomarker to refine renal prognosis. Interestingly, serum complement C4 was not associated with AAV prognosis, also supporting the fact that in humans, complement activation is rather mediated by cAP activation.

In an MPO-ANCA murine model, C5a and its receptor C5aR/CD88 have been evidenced as critical players in the development of NCGN in several studies [9–11]. The complement system can be engaged by activation of the classic, lectin and/or alternative pathway, that all lead to the conversion of C3 into C3a and C3b. C3b allows for the formation of C5 convertase that cleaves C5 into C5a and C5b and the assembly of the membrane attack complex (MAC) C5b-9 [23]. In the murine model of AAV, the knockout of C5 or Factor B, but not of C4 or C6 abrogates NCGN development, thus suggesting that cAP, and not the classic complement pathway or MAC, are implicated in tissue injury [9, 11, 12].

Consequently, several studies have been conducted in humans to investigate the potential role of the complement system in ANCA-associated glomerulonephritis [13, 14, 16]. These studies focused on histologic analysis of AAV glomerulonephritis and demonstrated, in contrast to what was previously thought, that immunoglobulins and/or complement components are detected in a large proportion of biopsies, by using electron microscopy or immunofluorescence [13, 14, 16]. Interestingly, in the recent study by Hilhorst et al, deposits of properdine, a positive cAP regulator, were found in a large proportion of AAV-glomerulonephritis biopsies and associated with the amount of cellular crescents, suggesting specific activation of the cAP [16].

Despite evidence of histologic features of complement activation, it is worth noting that circulating activation of complement, assessed with classical serum markers (such as total C3 and C4), is usually absent in human AAV. However, supporting systemic cAP activation in AAV patients, Ghou et al demonstrated that patients with active AAV had higher circulating C3a and C5a levels and properdine consumption than patients with remittent disease or healthy subjects [15].

In contrast to previous studies, in our work serum C3 and C4 usually fell within the normal range. We believe that these discrepancies may be related to C3 measurement technique and to the choice of lower limit cut-off value. In an effort to test the association between C3 and AAV outcomes, we defined low and high C3 level groups of patients based on the median value of C3 observed in the whole cohort. We observed that low serum C3 level at diagnosis was strongly linked to patient mortality and the risk of end-stage renal disease. Our analysis showing low C3 level significantly associated with renal death after adjustment on serum creatinine at admission suggest that low C3 could represent an independent risk factor of renal prognosis. However, the size of the study population did not allow extensive statistical analysis and no definite conclusion can be drawn. The association between C3 level and AAV prognosis has also been reported in two very recent studies that included 30 and 38 AAV patients in whom serum C3 levels were available at AAV diagnosis [24, 25]. We did not observe any difference in serum C3 level according to AAV type or ANCA specificity, even though MPA and MPO-ANCAs tended to be more represented in the low C3 group compared with the high C3 group. In contrast to C3, we did not observe any difference in patient mortality according to C4 levels, suggesting that the classic complement pathway does not modulate patient prognosis. Interestingly, CRP levels tended to be lower in low C3 group as compared to high C3 group. Given that we did not observe any difference between groups according to AAV subtype, organ involvement or BVAS, this suggests that CRP decrease may be related to cAP activation. In support, we observed a significant correlation between serum C3 level and CRP level (data not shown). CRP has been shown to interact in vitro with some regulators of the cAP [26]. Whether such a mechanism occurs in AAV patients and results in CRP consumption remains to be analysed.

Importantly, our work highlighted a significant negative correlation between serum C3 levels and the percentage of glomerular crescents per biopsy. This observation suggests that serum C3 level may represent a specific biomarker of renal injury. Moreover, the confrontation to the histologic categories using the Berden classification showed that C3 level may help to refine renal prognosis [22]. Indeed, our analysis suggests that patients with high C3 level and crescentic or mixed categories have an excellent renal prognosis compared with patients with low C3 levels (100% vs 40.7% at 6 years, p = 0.046). In a recent study, Manenti et al demonstrated a dramatic renal prognosis in a subset of AAV patients with both low serum C3 levels and AAV glomerulonephritis associated with thrombotic microangiopathy (TMA) lesions [24]. This observation raises the question of genetic or acquired defects of cAP regulators, that could result in C3 consumption and the development of superimposed renal TMA lesions. We did not observe TMA lesions in our patients, however TMA was not specifically looked for in our work. In this view, analysing C3 levels longitudinally in AAV patients may provide preliminary answers.

As expected, immune and complement deposits were only of low intensity in the immunofluorescence study of renal biopsies. Deposits were observed randomly in 80% of patients, which is in line with recent reports [13, 14, 16]. Also in line with a recent study [24], we did not observe any association between serum C3 levels and deposits, but we did not assess specific renal complement deposits such as C3c, C4d, properdine or MAC.

The main limitation of our work is the size of the population, its retrospective design and the lack of assessment of complement degradation fragments. Moreover, a renal biopsy was not available in 11/45 patients at AAV diagnosis, thus limiting the histological analysis. Despite these limitations, we were able to demonstrate clearly that low serum C3 level at AAV diagnosis is associated with a higher mortality and worse renal prognosis. We propose that C3 level should be assessed in further work to determine whether its association with the renal histologic category could help refine long-term renal prognosis, especially in patients with crescentic or mixed categories. Given the lack of association between C4 and AAV prognosis, our study also suggests a predominant role of cAP in human AAV.

## Supporting Information

S1 Fig Death-censored renal survival according to C3 levels and ANCA specificity. (TIFF)

S2 Fig C3 level and dialysis interruption in patients needing dialysis at AAV diagnosis. Fourteen patients needed renal replacement therapy at AAV diagnosis. (A) Percentage of dialysis interruption according to low or high C3 level groups. (B) C3 levels in patients that recovered renal function or that stayed dialysis-dependent.
(TIFF)

S3 Fig Immunoglobulin and complement deposits on immunofluorescence study of renal biopsies according to C3 levels. (TIFF)

S1 File Ethic committee waiver. (PDF)
