# Supplementary figures and images for: Low Serum Complement C3 Levels at Diagnosis of Renal ANCA-Associated Vasculitis Is Associated with Poor Prognosis
Source: PLoS One. 2016 Jul 8;11(7):e0158871. doi: 10.1371/journal.pone.0158871 (PMC4938207; doi:10.1371/journal.pone.0158871)

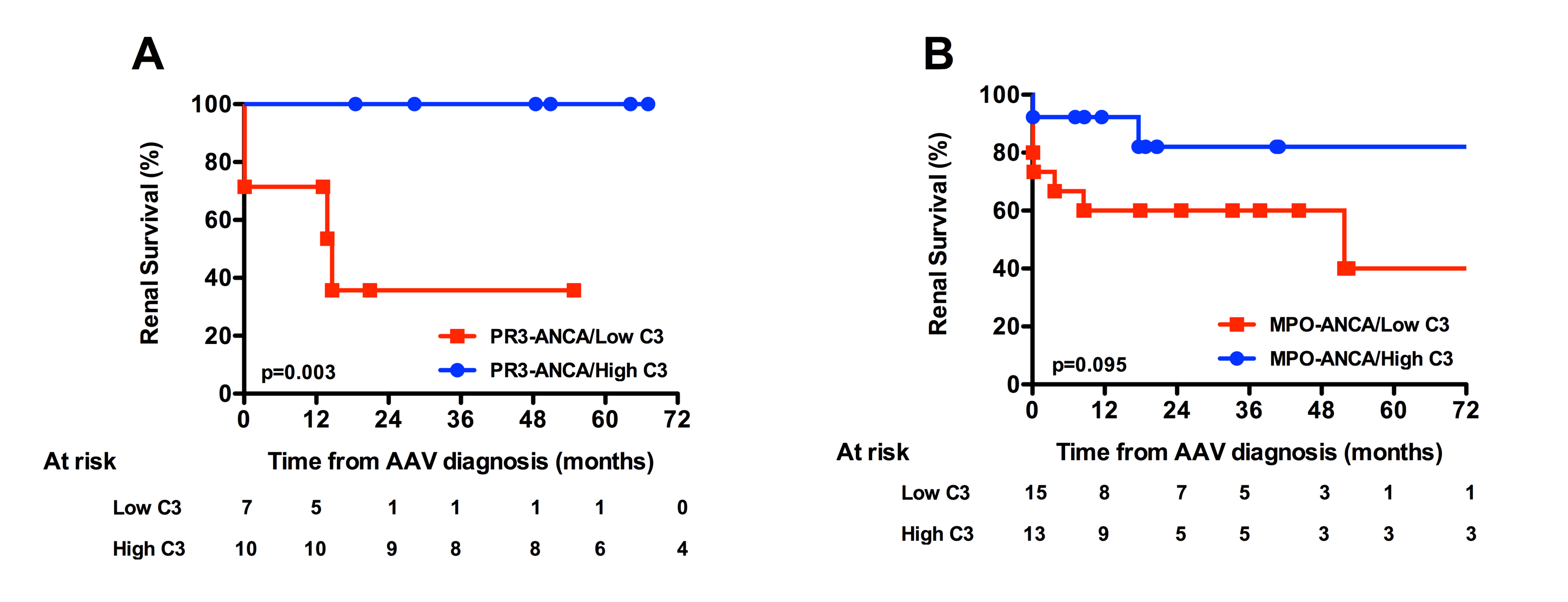

Supplement: S1 Fig — (TIFF) [file pone.0158871.s001.tiff]

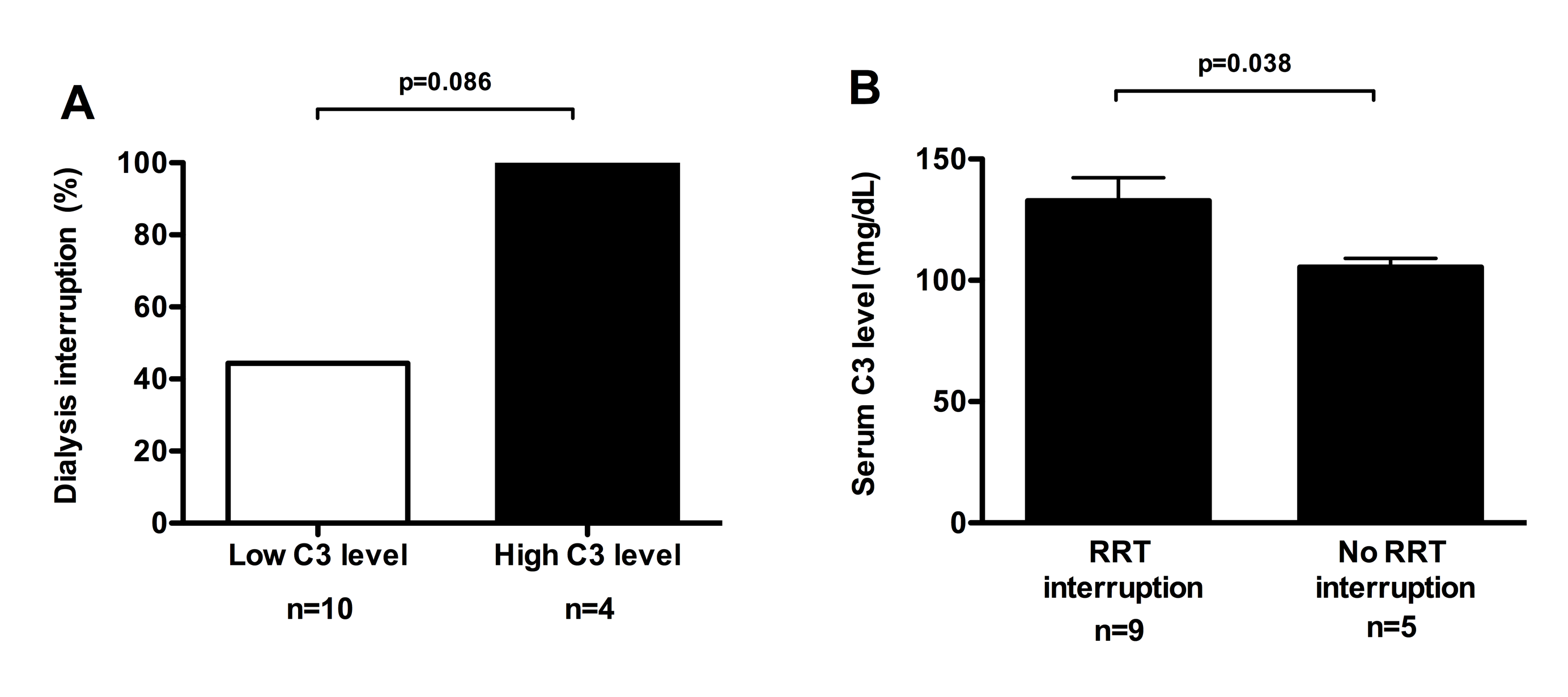

Supplement: S2 Fig — Fourteen patients needed renal replacement therapy at AAV diagnosis. (A) Percentage of dialysis interruption according to low or high C3 level groups. (B) C3 levels in patients that recovered renal function or that stayed dialysis-dependent. (TIFF) [file pone.0158871.s002.tiff]

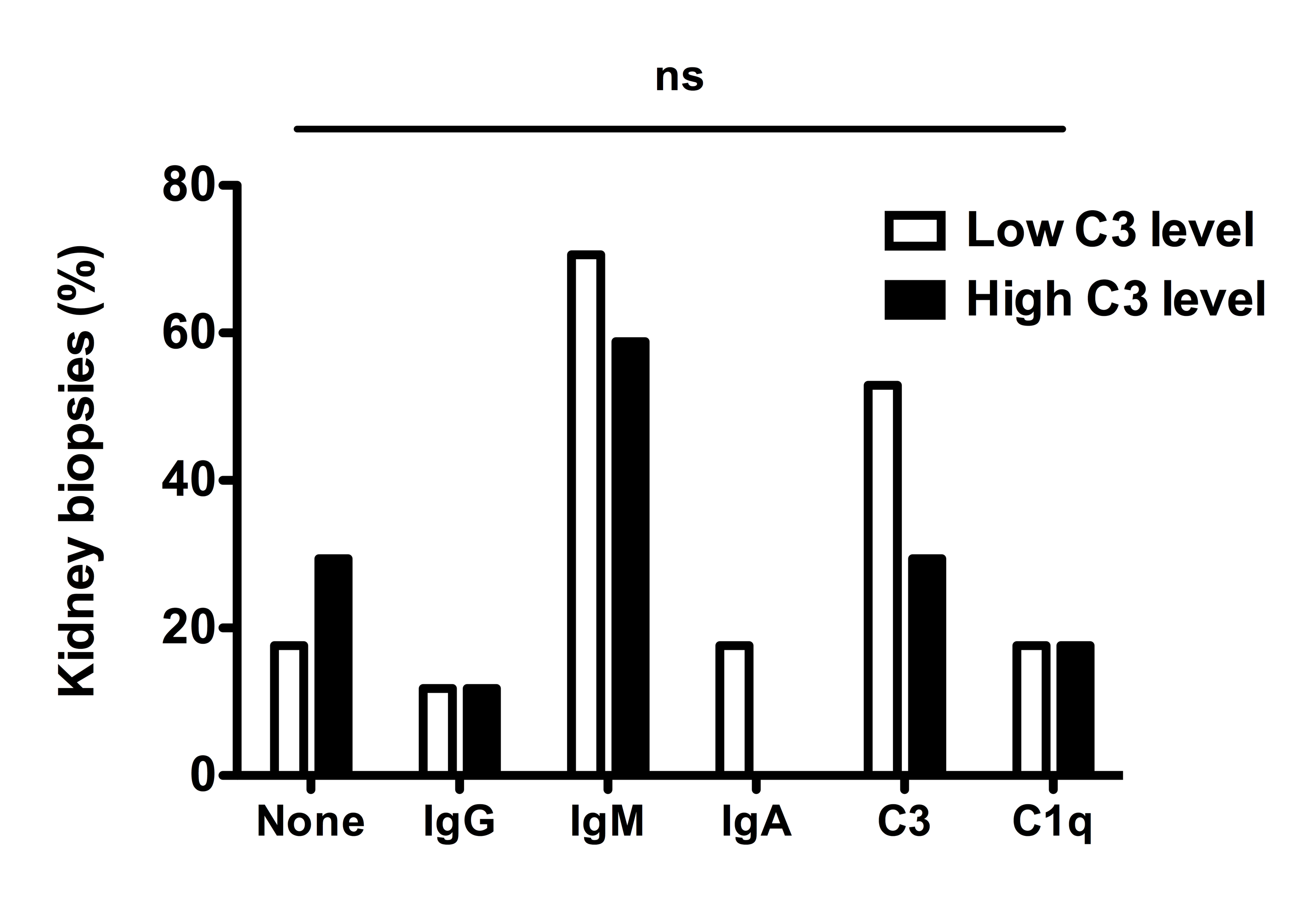

Supplement: S3 Fig — (TIFF) [file pone.0158871.s003.tiff]
