# Supplementary material for: Low Serum Complement C3 Levels at Diagnosis of Renal ANCA-Associated Vasculitis Is Associated with Poor Prognosis
Source: PLoS One. 2016 Jul 8;11(7):e0158871. doi: 10.1371/journal.pone.0158871 (PMC4938207; doi:10.1371/journal.pone.0158871)
Supplement: S1 File — (PDF) [file pone.0158871.s004.pdf]

FACULTE DE MEDECINE
ANGERS

CENTRE HOSPITALIER UNIVERSITAIRE
ANGERS

COMITE D'ETHIQUE

* * * * * *

Le Président
Professeur Jacques Dubin

Pr Jean-François Subra
Service de Néphrologie
CHU Angers

Angers, le 31 mai 2011

Cher Collègue,

Le Comité d'Ethique du Centre Hospitalier Universitaire d'Angers a examiné dans sa séance du **21 avril 2011** votre projet enregistré au comité sous le numéro **2011/06** et intitulé :
« Mise en évidence d'auto-anticorps anti-pentraxine 3 dans les vascularites à ANCA »

Dans le cadre d'une équipe de recherche INSERM U892, un nouvel autoanticorps a été mis en évidence dans le sang de patients souffrant de vascularite.
Le projet se propose de faire une recherche de cette autoanticorps sur une collection déjà existante.

Le comité d'éthique n'émet pas d'objection à la réalisation de ce travail sous réserve que la collection soit déclarée et inscrite dans le cadre d'un CRB.

Je vous prie de croire, Cher Collègue, en l'expression de mes sentiments les meilleurs.

Professeur Jacques Dubin

Maison de la Recherche Clinique
CHU Angers – 49933 Angers Cedex 9
Tél : 02 41 35 52 15 ou 02 41 35 79 22   Fax : 02 41 35 54 00
Email : jadubin@chu-angers.fr
